# MRSA Prevalence and Associated Risk Factors among Health-Care Workers in Non-outbreak Situations in the Dutch-German EUREGIO

**DOI:** 10.3389/fmicb.2016.01273

**Published:** 2016-08-22

**Authors:** Ricarda Sassmannshausen, Ruud H. Deurenberg, Robin Köck, Ron Hendrix, Annette Jurke, John W. A. Rossen, Alexander W. Friedrich

**Affiliations:** ^1^Institute of Hygiene, University Hospital MünsterMünster, Germany; ^2^Department of Medical Microbiology, University of Groningen, University Medical Center GroningenGroningen, Netherlands; ^3^Institute of Medical Microbiology, University Hospital MünsterMünster, Germany; ^4^CERTE-LvIGroningen, Netherlands; ^5^Department of Infectiology and Hygiene, Centre for Health North Rhine-WestphaliaMünster, Germany

**Keywords:** MRSA, decolonization, MRSA risk factors, personnel, staff, EurSafety health-net

## Abstract

Preventing the spread of methicillin-resistant *Staphylococcus aureus* (MRSA) in healthcare facilities is a major infection control target. However, only a few studies have assessed the potential role of healthcare workers (HCWs) for MRSA dissemination. To investigate the MRSA prevalence and the risk factors for MRSA colonization among HCWs, nasopharyngeal swabs were taken between June 2010 and January 2011 from 726 employees from nine acute care hospitals with different care levels within the German part of a Dutch-German border region (EUREGIO). The isolated MRSA strains were investigated using *spa* typing. The overall MRSA prevalence among HCWs in a non-outbreak situation was 4.6% (33 of 726), and was higher in nurses (5.6%, 29 of 514) than in physicians (1.2%, 1 of 83). Possible risk factors associated with MRSA colonization were a known history of MRSA carriage and the presence of acne. Intensive contact with patients may facilitate MRSA transmission between patients and HCWs. Furthermore, an accumulation of risk factors was accompanied by an increased MRSA prevalence in HCW.

## Introduction

Methicillin-resistant *Staphylococcus aureus* (MRSA) is one of the most important hospital-associated (HA) pathogens (Voss and Doebbeling, 1995). Although its prevalence among patients decreased in several European countries (e.g., UK, France, and Germany) during the last years, the MRSA prevalence increased in other countries (e.g., Norway and Poland; http://ecdc.europa.eu). In healthcare institutions, MRSA can be transmitted between patients, or through the hands, clothes, or equipment of healthcare workers (HCWs), and the environment (Haley et al., 1982; Hardy et al., 2006; Henderson, 2006). Furthermore, it has been reported that HCWs have been the source of MRSA outbreaks in several cases (Vonberg et al., 2006). In a systematic review of Albricht et al., in which 127 studies and outbreak reports published between January 1980 and March 2006 were reviewed, transmission of MRSA from HCWs to patients was likely in 63 of 68 (93%) studies as shown by genotyping (Albrich and Harbarth, 2008). The average MRSA prevalence among HCWs in these 127 studies was 4.6%, with a broad range from 0 to 59% (95% CI 1.0–8.2%) between countries and institutions. Notably, the MRSA prevalence rates were found to be higher in endemic situations (8.1%) compared to outbreak situations (3.9%). However, it has to be stated that different designs of the studies included limit the informative value of this review (Albrich and Harbarth, 2008).

The implementation of MRSA screening among HCWs has been shown to have a positive impact on outbreak management (Peters et al., 1999; Blok et al., 2003; Ben-David et al., 2008). Hence, infection control guidelines from many countries, including Germany, recommend a systematic screening of HCWs in the case of an acute outbreak of MRSA (Peters et al., 1999, 2014). In the Netherlands, having one of the lowest MRSA prevalence rates in Europe (http://ecdc.europa.eu), MRSA screening of HCWs is not conducted regularly, but in addition to outbreak cases, the national “Search-and-Destroy” strategy (www.wip.nl) recommends screening for further risk groups among personnel, such as staff returning from work in healthcare institutions abroad (Blok et al., 2003).

However, the “Search-and-Destroy” strategy remains subject of discussion (Hawkins et al., 2011; Hill, 2011) due to the lack of reliable prevalence data and an insufficient differentiation between stable and transient MRSA carriage. To the best of our knowledge, only a few studies have focused on the prevalence of MRSA colonization among HCWs in non-outbreak situations and the risk factors for stable and transient MRSA carriage in Germany. Furthermore, none of these studies involved HCWs from more than one hospital (Witte et al., 2005; Kaminski et al., 2007; Reich-Schupke et al., 2010). Therefore, the aim of the present study was to investigate the MRSA prevalence among HCWs in several hospitals in non-outbreak situations in the German part of a Dutch-German border region (“EUREGIO”) within the Dutch-German prevention network EurSafety Health-net (www.eursafety.eu) and assess data on (including non-occupational-related) risk factors for MRSA colonization among hospital staff.

## Materials and methods

### Participants

All hospitals in the German part of the EUREGIO were asked to participate in this study, and representatives of them were informed at the annual meeting of the German EUREGIO within the Dutch-German prevention network EurSafety Health-net. They discussed the study in their, respectively, hospital committees before the hospitals decided to participate in the study. Participation was voluntary for all HCWs and the directorate had to accept participation.

Between June 2010 and January 2011, nine acute care hospitals with different care levels within the German part of a Dutch-German EUREGIO participated in this study; two hospitals of basic care (No. 5 and 8), four hospitals of secondary care (No. 1, 4, 6, and 7) and three specialized clinics (No. 2, 3, and 9). The number of staff members within the hospitals ranged from 33 to 1640 and their capacities from 20 to 653 beds (Table 1). The participating hospitals had a total capacity of 2249 beds and employed 5264 staff members, including 667 physicians and 2325 nurses. In this study, both the medical staff and employees working outside direct patient-care, such as administration, maintenance, and cleaning, were encouraged to participate, and a questionnaire was used to obtain the needed information from the participants. Overall, 726 employees participated in this study (83 physicians, 514 nurses, 109 other staff members, and 20 participants of unknown professional group), covering 12% of the hospitals physicians and 22% of the nurses, respectively. As shown in Table 1, the study's coverage of medical staff varies between 9 and 91% between the different hospitals.

**Table 1:** **Overview of the participating hospitals and its personnel coverage shown as different professional groups**.

**Hospital**	**Beds (n)**		**Physicians**	**Nurses**	**Total medical staff**	**Others**	**Unknown**	**Total staff**
1	282	Total (n)	72	347	419	346		765
		Study (n%)	15 (21)	54 (16)	69 (17)	31 (9)	2	102 (13)
2	20	Total (n)	11	20	31	3		34
		Study (n%)	11 (100)	14 (70)	25 (81)	2 (67)	5	32 (94)
3	20	Total (n)	10	33	43	4		47
		Study (n%)	6 (60)	33 (100)	39 (91)	4 (100)	1	43 (92)
4	582	Total (n)	231	653	884	756		1640
		Study (n%)	5 (2)	81 (12)	86 (10)	5 (1)	0	91 (6)
5	271	Total (n)	63	210	273	212		485
		Study (n%)	29 (46)	183 (87)	212 (78)	63 (30)	1	276 (57)
6	405	Total (n)	135	484	619	469		1088
		Study (n%)	2 (2)	52 (11)	54 (9)	3 (1)	0	57 (5)
7	361	Total (n)	95	387	482	378		860
		Study (n%)	2 (2)	58 (15)	60 (12)	0 (0)	0	60 (7)
8	185	Total (n)	37	120	157	37		194
		Study (n%)	6 (16)	19 (16)	25 (16)	0 (0)	6	31 (16)
9	123	Total (n)	13	84	97	68		165
		Study (n%)	7 (54)	20 (24)	27 (28)	1 (2)	5	33 (20)

### Survey of risk factors

The participants' risk factors were collected using a standardized paper-based questionnaire (Supplementary Material). The following potential risk-factors for MRSA colonization were surveyed: history of MRSA carriage, profession, contact with MRSA carriers in a professional or private setting (with and without protective clothing), involvement in home-care of relatives, former professional occupation in a country known to be endemic for community-associated MRSA (CA-MRSA), dermatosis/diseases of the skin, chronic inflammatory bowel diseases (IBD), diseases of the upper respiratory tract, antibiotic therapy within the last 6 months, acute diseases (at the time of risk factor assessment), inpatient treatment within the last 6 or 12 months, contact with domestic or farm animals, and consumption of raw meat within the last 12 h. All questionnaires were processed using random pseudonyms that could only be decoded by the duty hygiene officer or the company medical officer.

### Nasopharyngeal swabs

HCWs can be MRSA positive after a working shift due to short-term contact with patients (transient MRSA carriers), while other HCWs are MRSA carriers for longer than 24 h (stable MRSA carriers) (Cookson et al., 1989; van Cleef et al., 2011). To differentiate between transient and stable MRSA carriers, swabs were taken twice on different days from all HCWs. Nasopharyngeal swabs were collected by trained personnel on Monday after an off-work weekend and subsequently on Wednesday before starting to work. To minimize the risk of considering a stable carrier as transient due to false-negative swabs, a third validation swab was collected in cases of discrepant results between the first and second sample. If the validation swab was positive, the HCW was considered to be a stable MRSA carrier, and if the validation swab was negative, the participant was regarded as a transient carrier. In addition, as skin colonization influenced decolonization-strategy, skin swabs (from the axilla and the groin) were taken from stable carriers immediately after being identified as such.

### MRSA isolation and *spa* typing

Swabs were applied to chromogenic media (BioMérieux, Nürtingen, Germany) the same day as the swab was collected and incubated for 48 h at 37°C. *S. aureus* colonies were tested for antibiotic resistance using the Vitek 2 (BioMérieux, Nürtingen, Germany) and the presence of the MRSA-specific penicillin binding protein 2a was tested by a latex-agglutination-test (Oxoid, Cambridge, UK). Every first MRSA isolate was further characterized using *S. aureus* protein A gene (*spa*) typing as described by Mellmann et al. (2008). Analysis was performed using the Staph Type™ software (Ridom GmbH, Münster, Germany) (Mellmann et al., 2007).

### Decolonization

If HCWs were identified as stable MRSA carriers, a decolonization protocol was started. They were asked to use mupirocin ointment intranasally and to gurgle with an octenidine-based solution, both thrice a day during a period of 6 days (Friday to Wednesday or Wednesday to Monday) and to attend a healthcare professional during the decolonization period. Moreover, all MRSA carriers were advised to daily change and wash clothes and bed-linen during the decolonization therapy. Those participants with skin colonization (additionally to nasopharyngeal colonization), as indicated by positive inguinal or axillary swabs, were also asked to daily wash themselves with octenidine-based soap.

### Statistical analysis

Data processing was performed using Microsoft Excel (Microsoft Corp., Redmont, USA) and statistical analyses were conducted with PASW Statistics (IBM Corp., Amonk, USA). Due to the small case numbers, statistical significance of risk factors was calculated in a univariate analysis with Fisher's exact test and *p* < 0.05 were regarded to be significant. To identify independent risk-factors, a multivariate analysis was performed using multiple logistic regression for all variable with *p* < 0.2 in univariate analysis and *p* < 0.05 were considered to be significant.

### Ethics

This study of screening HCW was approved by the Medical Chamber's of Westfalen-Lippe and Medical Faculty of the University of Münster's research ethic committee (2006-268-f-S). In all participating hospitals, the responsible member of the board of directors agreed to conduct the study. The employees participated in this study on a voluntary basis and written informed consent was obtained from all participants in accordance with the Declaration of Helsinki after the study's principal investigator and local infection control manager held meetings to inform the participant about the study.

## Results

### Swabs

At least two nasopharyngeal swabs from each of the 726 HCWs were collected. In 21 HCWs, both swabs were MRSA positive, and in 12 HCWs only one swab was positive for MRSA. A third “validation”-swab was required for these 12 HCWs. As five participants refused to give permission to take a third swab, and started already with decolonization, persistence of colonization could not be determined for these HCWs (Table 2).

**Table 2:** **MRSA colonization among healthcare-workers in the EUREGIO screened twice for nasopharyngeal carriage**.

**Initial swab**			Validation swab^*^		
Result^**^	**Number**	**Percentage**	**Participation**	**Positive swab**	Negative swab^***^
(−/−)	693	95.45	NA	NA	NA
(+/+)	21	2.89	NA	NA	NA
(−/+)	4	0.55	2 of 4	0	2
(+/−)	8	1.11	5 of 8	2	3
Total	726	−	−	−	−

* *Validation swab to confirm or exclude stable MRSA colonization among HCWs with discordant results of initial two nasopharyngeal swabs*;

** *MRSA test result (−/−), negative in two nasopharyngeal swabs; (+/+), MRSA detected in two nasopharyngeal swabs; (−/+) and (+/−), MRSA detected in one of two nasopharyngeal swabs, a validation swab was taken in these cases*;

*** *NA, not applicable*.

### MRSA prevalence

In 33 of the 726 HCWs at least one swab was MRSA positive. Consequently, the MRSA prevalence was 4.6%. Two of the seven participants with discrepant swab results had a positive validation swab. Thus, 23 HCWs appeared to be stable carriers, whereas 5 HCWs were transient carriers resulting in a prevalence of 3.2% for stable carriers and 0.7% for transient carriers. In total, 17.9% of the detected cases of MRSA colonization were transient according to the applied definition.

**Figure 1:**
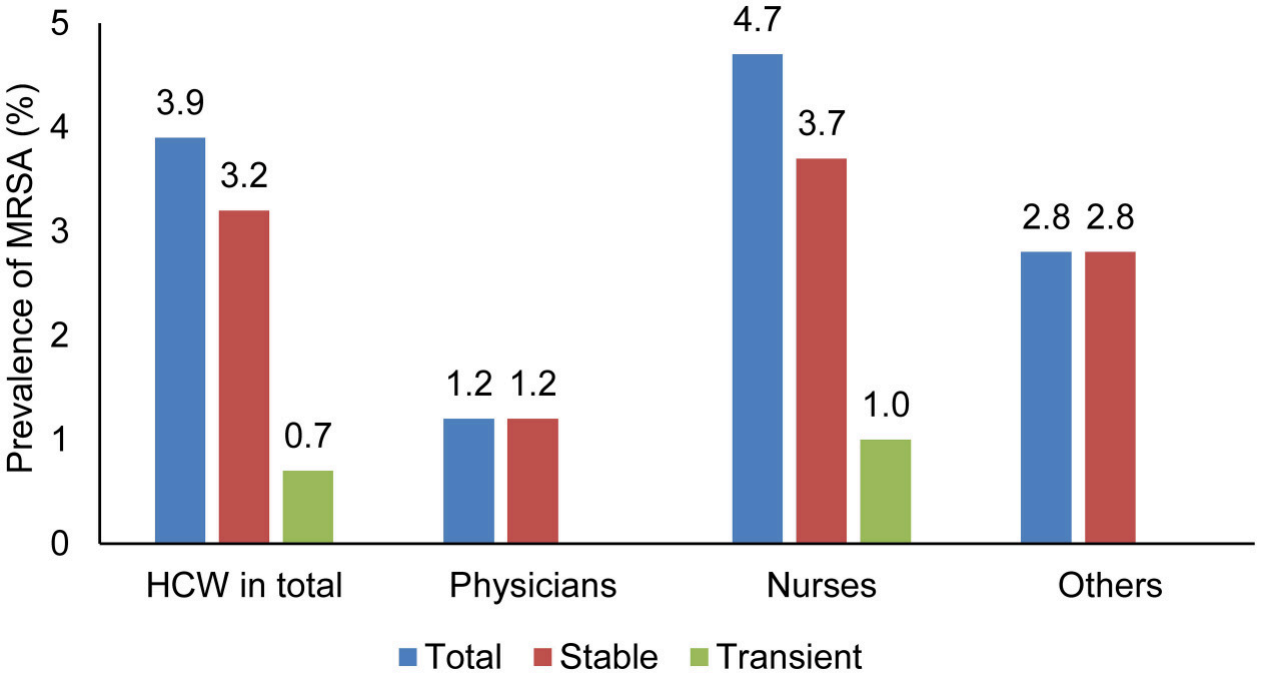
**MRSA prevalence among HCWs in different professions and the differentiation between stable and transient MRSA carriers**.

The prevalence of MRSA differed for the type of healthcare profession. Among physicians, the MRSA prevalence was lower (1.2%, 1 of 83) compared to nurses (5.6%, 29 of 514). In nurses, stable MRSA carriage was found in 3.7% (19 of 514) of the cases, while transient MRSA carriage was observed in 1.0% (5 of 514) (Figure 1). Interestingly, stable MRSA positivity was observed in 2.8% (3 of 109) of other staff members who had no direct contact with patients.

### *Spa* typing

Nine different *spa* types were observed among the 34 MRSA isolates [t003 (*n* = 13), t032 (*n* = 11), t151 (*n* = 3), t004 (*n* = 1), t020 (*n* = 1), t022 (*n* = 2), t179 (*n* = 1), t2261 (*n* = 1), and t6015 (*n* = 1)]. One HCW was colonized with two genetically non-related MRSA strains (*spa* type t004 and t032). Most frequently, typical HA-MRSA strains with *spa* type t003 (38%) and t032 (32%) were observed. The observation of these *spa* types is in accordance with the most prevalent *spa* types found among patients in the study region (Köck et al., 2009).

### Risk factors for MRSA colonization

For analysis of the risk factors for MRSA colonization in HCWs, all participants with at least one positive swab were included. The statistical analysis of the risk factors is shown in Table 3. Previous MRSA colonization (*p* = 0.01, CI = 2.2–24.8), acne (*p* = 0.049, CI = 1.008–19.07) and chronic IBD (*p* = 0.005, CI = 3.66–1457.33) were significantly associated with MRSA carriage among HCWs in the multivariate analysis.

**Table 3:** **Risk-factor analysis for MRSA colonization in HCWs**.

**Risk factor**	**MRSA negative (*n* = 693)**	**MRSA positive (*n* = 33)**	**Fisher's exact test**			Logistic regression^*^		
			**Odds ratio**	*p* (FE)^*^	**95%CI (FE)**	**Odds ratio**	***p* (FE)**	**95%CI (FE)**
Physicians	82	1	0.23	0.162	0.31–1.72	0.7	0.7	0.63–7.79
Nurses	485	29	3.11	0.030	1.08–8.96	2.81	0.15	0.69–11.43
Staff without direct contact with patients	109	3	0.55	0.460	0.17–1.85	–	–	–
Unknown profession	20	0	0.97	1.000	0.96–0.98	–	–	–
Previous MRSA carrier	16	5	7.56	0.002	2.58–22.09	7.44	0.01	2.2–24.8
Occupation on ICU	102	9	2.17	0.077	0.98–4.81	2.05	0.12	0.84–4.96
Occupation with new-born	16	1	1.32	0.551	0.17–10.28	–	–	–
Occupation in foreign countries	3	0	1.00	1.000	1–1	–	–	–
Contact with MRSA carriers without protective clothing	68	4	1.31	0.549	0.45–3.84	–	–	–
Contact with MRSA carriers with protective clothing	227	16	1.92	0.088	0.95–8.87	1.04	0.94	0.39–2.7
Home-care of relatives	14	2	3.13	0.162	0.68–14.37	4.28	0.097	0.77–23.76
Contact with MRSA carriers at home	7	2	6.32	0.059	1.26–31.7	6.63	0.063	0.9–48.91
Atopic dermatitis	33	3	2.00	0.221	0.58–6.90	–	–	–
Paronchyia	1	0	1.00	1.000	1–1	–	–	–
Acne	13	3	5.23	0.032	1.42–19.34	4.38	0.049	1.008–19.07
Otitis	4	1	5.38	0.208	0.59–49.55	–	–	–
Open wounds	5	0	1.00	1.000	1–1	–	–	–
Chronic inflammatory bowel disease	1	1	21.63	0.089	1.32–353.62	73.12	0.005	3.66–1457.33
Sinusitis	23	0	0.97	0.619	0.96–0.98	–	–	–
Diabetes mellitus	8	1	2.68	0.344	0.325–22.05	–	–	–
Rhinitis	25	2	1.72	0.350	0.39–7.61	–	–	–
Other chronic diseases	64	3	0.98	1.000	0.29–3.3	–	–	–
Antibiotics	81	4	1.04	1.000	0.36–3.04	–	–	–
Hospitalization for >24 h in the last 6 months	23	1	0.91	1.000	0.12–6.95	–	–	–
Hospitalization for >24 h in the last 6–12 months	24	0	0.97	0.620	0.95–0.98	–	–	–
Hospitalization for >24 h in a foreign country	0	1	0.45	0.715	0.6–3.37	–	–	–
Contact with farm animals	27	2	1.59	0.384	0.36–7	–	–	–
Thereof contact with pigs	17	1	1.24	0.570	0.16–9.63	–	–	–
Contact with domestic animals	265	16	1.52	0.270	0.76–3.06	–	–	–
Contact with raw meat within last 12 h	62	5	1.82	0.218	0.678–4.88	–	–	–
Acute illness	32	3	2.07	0.209	0.6–7.13	–	–	–

* *Risk factors with p < 0.2 in univariate analysis (Fisher's exact test) were included in logistic regression*.

**Figure 2:**
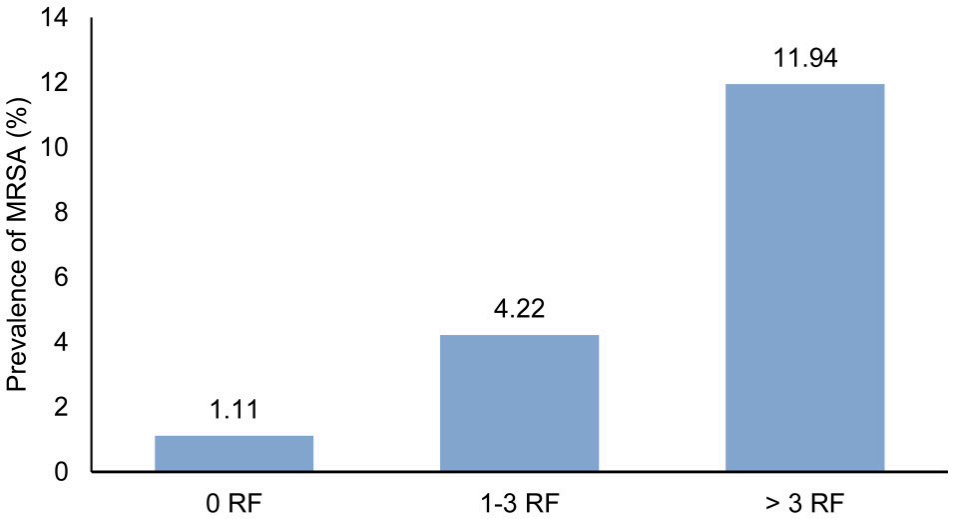
**Prevalence of MRSA in HCWs grouped by the number of the participants' overall risk factors (RF)**.

For the evaluation of risk factor groups, all risk factors with an odds ratio >1 in the univariate analysis were taken into account. In order to analyze a possible impact of the accumulation of risk factors, all participants were grouped in three categories: 0, 1, to 3, or >3 risk factors. In total, 12.4% of the staff members were free of all risk factors analyzed, for the majority of participants (78.4%) 1–3 risk factors were applicable, and 9.2% of the HCWs were associated with more than three risk factors. The differences in MRSA prevalence according to the risk factor group are shown in Figure 2.

### Decolonization

Only stable MRSA carriers were decolonized. Five participants who did not participate in the validation screening were excluded from this analysis, and two HCWs wished to be treated outside the study. So, 21 HCWs participated in the controlled decolonization. Two were colonized with MRSA on the skin and underwent the entire decolonization measures including skin washes. Nineteen HCWs (90%) underwent nasopharyngeal decolonization only. All control swabs after the decolonization attempt were negative. Six of the 21 HCWs (29%), among those the HCWs with skin-colonization, agreed on re-swabbing after 1 month, while nine HCWs (39%) were re-swabbed after 6 months. All of these control swabs were negative and confirmed the initial test result.

## Discussion

A 4.6% MRSA prevalence was observed among HCWs in the present study, which was lower than described by Albrich et al. (8.1% outside outbreak situations in several different countries), but similar to the results of Kaminski et al. (4.6% inside outbreak situations) in Germany in 2001 and 2002 (Kaminski et al., 2007; Albrich and Harbarth, 2008). Intensive training of healthcare personnel and an increased public awareness toward MRSA in the framework of the EUREGIO MRSA-net (Friedrich et al., 2008) may have contributed to the successful prevention of MRSA transmission in- and out-side outbreak situations.

The MRSA prevalence among HCWs in the EUREGIO is high compared to the MRSA prevalence observed among patients upon hospital admission. As Köck et al. have shown, MRSA admission prevalence rates in patients are approximately 2.5 times lower than in HCWs (Köck et al., 2010). In consequence, HCWs are at higher risk for MRSA colonization than the general population due to the constant contact exposure to known or unknown MRSA-positive patients. This is also underpinned by the typing results of MRSA isolated from HCWs. In this study, mainly *spa* types t003 and t032, associated with the classical German healthcare-associated MRSA lineages CC5 and CC22, respectively, were observed. Transmission between patients and HCWs is likely, as these *spa* types also predominated among regional patients at the same time (Köck et al., 2013). By contrast, livestock-associated MRSA (LA-MRSA), e.g., *spa* type t011 and t034, which are the third and fourth most prevalent *spa* types among patients in the German part of the EUREGIO (Köck et al., 2011, 2013), could not be detected at all in HCWs. Since all MRSA isolates from patients in the EUREGIO are characterized, *spa* types observed in HCWs and patients in the corresponding hospital could be compared. The most prevalent *spa* types observed among HCWs were: t003 (38.2%), t032 (32.4%), t151 (8.8%), t022 (5.9%), t004, t020, t179, t2261, and t6015 (each 2.9%), while *spa* types t003 (24.7%), t032 (19.1%), t011 (13.1%), t034 (9.2%), and t020 (2.5%) were most prevalent in patients in the EUREGIO. This discrepancy of the distribution of *spa* types in patients and HCWs can be explained by the observations that the risk for LA-MRSA-colonization is 168 times higher for people who work daily with pigs than for family members living on a farm and that transmission of LA-MRSA between humans outside farms is limited (Cuny et al., 2009). The reason therefore is still unknown. Among the HCWs screened in this study, only 4% had contact with livestock.

In the different professional groups, our observations confirm the results found in previous studies, which showed a higher MRSA prevalence in nurses compared to medical doctors (Kaminski et al., 2007; Albrich and Harbarth, 2008). HCWs are likely to act more frequently as vectors, rather than being the main source of MRSA transmission. Furthermore, MRSA can be transmitted both from nurses to patients and from patients to nurses. Although colonized HCWs appeared to be most often transiently carriers, they may become stable carriers if they have sinusitis, or chronic dermatitis, leading to MRSA transmission over a longer time period (Dulon et al., 2014). The discussion on who is transmitting MRSA to whom has also been discussed in relation with MRSA observed in (pet) animals and possible transmission to humans. A Dutch study showed that a dog living in close contact with a nurse became colonized with MRSA resulting in recurrent MRSA colonization of the nurse (van Duijkeren et al., 2004). In addition, a narrative review concluded that both dogs and cats can serve as vectors for MRSA transmission (Bramble et al., 2011). Consequently, it can be hypothesized that close physical contact with patients, e.g., washing and dressing, contributes to a higher MRSA transmission rate between patients and HCWs. The high MRSA prevalence among employees working in non-patient related activities (2.8%), also higher than in medical doctors (1.2%), seems to contradict this assumption. A possible explanation for this could be that non-patient related activities, e.g., cleaning personnel, is exposed to patients and especially patient fluids and materials in toilets and bathrooms, but may be less informed about the protocols to prevent indirect MRSA transmission. Furthermore, two of the three MRSA-positive samples from people with non-patient related activities in this study were either in contact with MRSA-positive relatives, or cared for relatives at home. Both these risk factors had a high odds ratio in the univariate analysis, but were not statistically significant risk factors for MRSA colonization.

It would be interesting to know the MRSA prevalence among surgeons and the staff from surgical wards. Unfortunately, the present study was underpowered to detect such possible differences. This was due to the fact that collection of data on the profession of the participants was not mandatory. From 85 participants, it is known that they were medical doctors. Of these 85 medical doctors, it was possible in only 39 cases to distinguish between surgeons and non-surgeons, and analyses resulted in 15 surgeons and 24 non-surgeons. Only one of these 15 surgeons was MRSA positive.

*S. aureus* colonization may be dependent on both host and bacterial factors. Furthermore, it has been reported that after decolonization, stable carriers often become re-colonized with the same *S. aureus* strain, whereas non-carriers resist experimental colonization. Long-term carriers of *S. aureus* were reported to carry *S. aureus* between 70 days and 8 years (Brown et al., 2014). Two MRSA swabs were taken from HCWs in order to differentiate between transient and stable carriage. Previously, it has been shown that transient carriage is especially found when HCWs are swabbed during or after their shifts (Cookson et al., 1989). Even though HCWs were sampled before starting their shifts, we observed heterogeneous swab results, indicating transient carriage among 5 of the 28 MRSA-positive HCWs. Besides transient carriage, heterogeneous results may also be the consequence of false-negative results in one swab of an actual stable carrier due to a sampling error, the less than 100% sensitivity of the nasopharyngeal swabs (Kunori et al., 2002), or the lack of the use of a semi-selective broth before application of the swabs on chromogenic media (Bocher et al., 2010) as used in the present study. To exclude false-negative results, a third validation swab was taken. This validation swab confirmed negative swab results, and thereby a stable carriage rate of 69.7%. So, 17.9% of all MRSA carriers proved to be transient carriers, and we conclude that differentiation between transient and stable carriers seems appropriate and reduces the number of HCWs who needed to be included in decolonization therapies. By contrast, abdication of differentiation might overestimate actual MRSA prevalence in HCWs and lead to unnecessary decolonization.

According to the multivariate analysis, a positive anamnesis of MRSA carriage, acne and chronic IBD were possible risk factors for MRSA colonization in HCWs. As the latter only refers to one MRSA positive HCW and one MRSA negative HCW, its risk impact is not clear and should be confirmed using a larger cohort of individuals suffering from IBD. The other two possible risk factors confirmed observations in previous studies (Albrich and Harbarth, 2008). The fact that a positive anamnesis is a highly significant (*p* = 0.01) risk factor, encourages attempts for long-term control of MRSA-positive HCWs. Although we have shown that all HCWs could be decolonized initially, a positive MRSA anamnesis still increases the risk for re-colonization or resurge of suppressed colonization. The reason for re-colonization/resurge is unclear, but a possible vulnerability for MRSA colonization and frequent long-term failure of topical decolonization therapies might contribute to it (Ammerlaan et al., 2009). As shown before, chronic skin diseases, such as acne in the present study, are known risk factors for MRSA colonization (Berthelot et al., 2003). As the number of individuals included in our study is rather small, the factors identified as possible risk factors should be confirmed in a larger group.

The drawback of this study was the great variation in the participation rate of HCWs among the hospitals, e.g., in hospital no. 6 only 9% of the HCWs participated, whereas in hospital no. 3 the participation rate was 91%. This variation in the participation rate of HCWs was due to different factors. Our aim was to screen approximately 100 persons per hospital, or less if the hospital was smaller. We argued with hospital directorates that not less than 100 persons per hospital should participate in order to enlarge the acceptability of the study. The directorate was concerned that the hospital would be understaffed because of the expected high rates of MRSA-positive employees and the fact that MRSA-positive employees had to be decolonized and could not work for a few days. In the future, well-organized campaigns focusing on the benefit for the hospital to participate in studies, such as ours, may take away their concern. Furthermore, the number of participants was also dependent on the commitment and the networking of the representatives of the hospitals. If these representatives were committed and networked well, which is much easier in smaller hospitals, more employees could be screened. To the best of our knowledge, there were no studies in Germany in 2012 in which more physicians were included for MRSA screening.

In this study, the MRSA prevalence among HCWs in a non-outbreak situation is not as high as observed in other studies, but still higher compared to the MRSA prevalence in patients at hospital admission. We showed that 70% of HCWs were stable MRSA carriers, whereas 30% “lost” MRSA in control swabs taken without decolonization attempts and indicating transient colonization/contamination. Since a higher MRSA prevalence in nurses compared to medical doctors, it can be suggested that close physical contact with patients contributes to a higher MRSA transmission rate between patients and HCWs.

## Author contributions

RS, RD, RK, RH, AJ, JR, and AF planned the study, collected and analyzed the data and wrote the manuscript.

## Funding

This study was supported by the Interreg IVa-funded projects EurSafety Heath-net (III-1-02 = 73) and SafeGuard (III-2-03 = 025), part of a Dutch-German cross-border network supported by the European Commission, the German Federal States of Nordrhein-Westfalen and Niedersachsen, and the Dutch provinces of Overijssel, Gelderland, and Limburg and by the German Ministry of Education and Research (MedVet-Staph No. 01KI1014A).

### Conflict of interest statement

The authors declare that the research was conducted in the absence of any commercial or financial relationships that could be construed as a potential conflict of interest.

## References

[B1] Albrich W. C. Harbarth S. (2008). Health-care workers: source, vector, or victim of MRSA? Lancet Infect. Dis. 8, 289–301. 10.1016/S1473-3099(08)70097-5 18471774

[B2] Ammerlaan H. S. Kluytmans J. A. Wertheim H. F. Nouwen J. L. Bonten M. J. (2009). Eradication of methicillin-resistant *Staphylococcus aureus* carriage: a systematic review. Clin. Infect. Dis. 48, 922–930. 10.1086/597291 19231978

[B3] Ben-David D. Mermel L. A. Parenteau S. (2008). Methicillin-resistant *Staphylococcus aureus* transmission: the possible importance of unrecognized health care worker carriage. Am. J. Infect. Control 36, 93–97. 10.1016/j.ajic.2007.05.013 18313510

[B4] Berthelot P. Grattard F. Fascia P. Fichtner C. Moulin M. Lavocat M. P.. (2003). Implication of a healthcare worker with chronic skin disease in the transmission of an epidemic strain of methicillin-resistant *Staphylococcus aureus* in a pediatric intensive care unit. Infect. Control Hosp. Epidemiol. 24, 299–300. 10.1086/502208 12725361

[B5] Blok H. E. Troelstra A. Kamp-Hopmans T. E. Gigengack-Baars A. C. Vandenbroucke-Grauls C. M. Weersink A. J.. (2003). Role of healthcare workers in outbreaks of methicillin-resistant *Staphylococcus aureus*: a 10-year evaluation from a Dutch university hospital. Infect. Control Hosp. Epidemiol. 24, 679–685. 10.1086/502275 14510251

[B6] Böcher S. Middendorf B. Westh H. Mellmann A. Becker K. Skov R.. (2010). Semi-selective broth improves screening for methicillin-resistant *Staphylococcus aureus*. J. Antimicrob. Chemother. 65, 717–720. 10.1093/jac/dkq001 20130023

[B7] Bramble M. Morris D. Tolomeo P. Lautenbach E. (2011). Potential role of pet animals in household transmission of methicillin-resistant *Staphylococcus aureus*: a narrative review. Vector Borne Zoonotic Dis. 11, 617–620. 10.1089/vbz.2010.0025 21142959 PMC3115421

[B8] Brown A. F. Leech J. M. Rogers T. R. McLoughlin R. M. (2014). *Staphylococcus aureus* colonization: modulation of host immune response and impact on human vaccine design. Front. Immunol. 4:507. 10.3389/fimmu.2013.00507 24409186 PMC3884195

[B9] Cookson B. Peters B. Webster M. Phillips I. Rahman M. Noble W. (1989). Staff carriage of epidemic methicillin-resistant *Staphylococcus aureus*. J. Clin. Microbiol. 27, 1471–1476. 2768437 10.1128/jcm.27.7.1471-1476.1989 PMC267597

[B10] Cuny C. Nathaus R. Layer F. Strommenger B. Altmann D. Witte W. (2009). Nasal colonization of humans with methicillin-resistant *Staphylococcus aureus* (MRSA) CC398 with and without exposure to pigs. PLoS ONE 4:e6800. 10.1371/journal.pone.0006800 19710922 PMC2728842

[B11] Dulon M. Peters C. Schablon A. Nienhaus A. (2014). MRSA carriage among healthcare workers in non-outbreak settings in Europe and the United States: a systematic review. BMC Infect. Dis. 14:363. 10.1186/1471-2334-14-363 24996225 PMC4094410

[B12] Friedrich A. W. Daniels-Haardt I. Köck R. Verhoeven F. Mellmann A. Harmsen D.. (2008). EUREGIO MRSA-net Twente/Munsterland - a Dutch-German cross-border network for the prevention and control of infections caused by methicillin-resistant *Staphylococcus aureus*. Euro Surveill. 13, 1–5. 18761882 10.2807/ese.13.35.18965-en

[B13] Haley R. W. Hightower A. W. Khabbaz R. F. Thornsberry C. Martone W. J. Allen J. R.. (1982). The emergence of methicillin-resistant *Staphylococcus aureus* infections in United States hospitals. Possible role of the house staff-patient transfer circuit. Ann. Intern. Med. 97, 297–308. 7114626 10.7326/0003-4819-97-3-297

[B14] Hardy K. J. Oppenheim B. A. Gossain S. Gao F. Hawkey P. M. (2006). A study of the relationship between environmental contamination with methicillin-resistant *Staphylococcus aureus* (MRSA) and patients' acquisition of MRSA. Infect. Control Hosp. Epidemiol. 27, 127–132. 10.1086/500622 16465628

[B15] Hawkins G. Stewart S. Blatchford O. Reilly J. (2011). Should healthcare workers be screened routinely for meticillin-resistant *Staphylococcus aureus*? A review of the evidence. J. Hosp. Infect. 77, 285–289. 10.1016/j.jhin.2010.09.038 21292349

[B16] Henderson D. K. (2006). Managing methicillin-resistant Staphylococci: a paradigm for preventing nosocomial transmission of resistant organisms. Am. J. Med. 119(6 suppl. 1), S45–S52. discussion: S62–S70. 10.1016/j.amjmed.2006.04.002 16735151

[B17] Hill S. F. (2011). Should healthcare workers be screened routinely for meticillin-resistant *Staphylococcus aureus*? J. Hosp. Infect. 79, 275. 10.1016/j.jhin.2011.05.019 21767895

[B18] Kaminski A. Kammler J. Wick M. Muhr G. Kutscha-Lissberg F. (2007). Transmission of methicillin-resistant *Staphylococcus aureus* among hospital staff in a German trauma centre: a problem without a current solution? J. Bone Joint Surg. Br. 89, 642–645. 10.1302/0301-620X.89B5.18756 17540751

[B19] Köck R. Becker K. Cookson B. van Gemert-Pijnen J. E. Harbarth S. Kluytmans J.. (2010). Methicillin-resistant *Staphylococcus aureus* (MRSA): burden of disease and control challenges in Europe. Euro Surveill. 15, 19688. 20961515 10.2807/ese.15.41.19688-en

[B20] Köck R. Brakensiek L. Mellmann A. Kipp F. Henderikx M. Harmsen D.. (2009). Cross-border comparison of the admission prevalence and clonal structure of meticillin-resistant *Staphylococcus aureus*. J. Hosp. Infect. 71, 320–326. 10.1016/j.jhin.2008.12.001 19201056

[B21] Köck R. Schaumburg F. Mellmann A. Köksal M. Jurke A. Becker K.. (2013). Livestock-associated methicillin-resistant *Staphylococcus aureus* (MRSA) as causes of human infection and colonization in Germany. PLoS ONE 8:e55040. 10.1371/journal.pone.0055040 23418434 PMC3572123

[B22] Köck R. Siam K. Al-Malat S. Christmann J. Schaumburg F. Becker K.. (2011). Characteristics of hospital patients colonized with livestock-associated meticillin-resistant *Staphylococcus aureus* (MRSA) CC398 versus other MRSA clones. J. Hosp. Infect. 79, 292–296. 10.1016/j.jhin.2011.08.011 22024921

[B23] Kunori T. Cookson B. Roberts J. A. Stone S. Kibbler C. (2002). Cost-effectiveness of different MRSA screening methods. J. Hosp. Infect. 51, 189–200. 10.1053/jhin.2002.1247 12144798

[B24] Mellmann A. Weniger T. Berssenbrügge C. Keckevoet U. Friedrich A. W. Harmsen D.. (2008). Characterization of clonal relatedness among the natural population of *Staphylococcus aureus* strains by using *spa* sequence typing and the BURP (Based Upon Repeat Patterns) algorithm. J. Clin. Microbiol. 46, 2805–2808. 10.1128/JCM.00071-08 18524961 PMC2519486

[B25] Mellmann A. Weniger T. Berssenbrügge C. Rothganger J. Sammeth M. Stoye J.. (2007). Based Upon Repeat Pattern (BURP): an algorithm to characterize the long-term evolution of *Staphylococcus aureus* populations based on *spa* polymorphisms. BMC Microbiol. 7:98. 10.1186/1471-2180-7-98 17967176 PMC2148047

[B26] Peters G. Becker K. Briesch H. Hergenröder H. Heudorf U. Just H. M. (2014). Empfehlungen zur Prävention und Kontrolle von Methicil-lin-resistenten *Staphylococcus aureus*-Stämmen (MRSA) in medizinischen und pflegerischen Einrichtungen. Bundesgesundheitsblatt 57, 696–732. 10.1007/s00103-014-1980-x 24987771

[B27] Peters G. Becker K. Kipp F. Heuck D. Nassauer A. Unger G. (1999). Empfehlung zur Prävention und Kontrolle von Methicillin-resistenten *Staphylococcus aureus*-Stämmen (MRSA) in Krankenhäusern und anderen medizinischen Einrichtungen Mitteilung der Kommission für Krankenhaushygiene und Infektionsprävention am RKI. Bundesgesundheitsblatt 42, 954–958.

[B28] Reich-Schupke S. Geis G. Reising M. Altmeyer P. Stücker M. (2010). MRSA in dermatology - prospective epidemiological study in employees and patients of a dermatological department of a university hospital. J. Dtsch. Dermatol. Ges. 8, 607–613. 10.1111/j.1610-0387.2010.07381.x 20184668

[B29] van Cleef B. A. Graveland H. Haenen A. P. van de Giessen A. W. Heederik D. Wagenaar J. A.. (2011). Persistence of livestock-associated methicillin-resistant *Staphylococcus aureus* in field workers after short-term occupational exposure to pigs and veal calves. J. Clin. Microbiol. 49, 1030–1033. 10.1128/JCM.00493-10 21227986 PMC3067751

[B30] van Duijkeren E. Wolfhagen M. J. Box A. T. Heck M. E. Wannet W. J. Fluit A. C.. (2004). Human-to-dog transmission of methicillin-resistant *Staphylococcus aureus*. Emerg. Infect. Dis. 10, 2235–2237. 10.3201/eid1012.040387 15663871 PMC3323405

[B31] Vonberg R. P. Stamm-Balderjahn S. Hansen S. Zuschneid I. Ruden H. Behnke M.. (2006). How often do asymptomatic healthcare workers cause methicillin-resistant *Staphylococcus aureus* outbreaks? A systematic evaluation. Infect. Control. Hosp. Epidemiol. 27, 1123–1127. 10.1086/507922 17006821

[B32] Voss A. Doebbeling B. N. (1995). The worldwide prevalence of methicillin-resistant *Staphylococcus aureus*. Int. J. Antimicrob. Agents 5, 101–106. 18611655 10.1016/0924-8579(94)00036-t

[B33] Witte W. Mielke M. Ammon A. Nassauer A. Wischnewski N. (2005). Fachtagung der AG Nosokomiale Infektionen am RKI zur Intensivierung der Umsetzung von Präventionsstrategien bei MRSA. Epid. Bull. 5, 31–38.

