# Supplementary material for: MRSA Prevalence and Associated Risk Factors among Health-Care Workers in Non-outbreak Situations in the Dutch-German EUREGIO
Source: Front Microbiol. 2016 Aug 22;7:1273. doi: 10.3389/fmicb.2016.01273 (PMC4993013; doi:10.3389/fmicb.2016.01273)
Supplement: Supplementary file 1 [file DataSheet1.DOCX]

Supplementary Material

MRSA Prevalence and Associated Risk Factors among Health-care Workers in Non-outbreak Situations in the Dutch-German EUREGIO

Ricarda Sassmannshausen, Ruud H. Deurenberg, Robin Köck, Ron Hendrix, Annette Jurke, John W.A. Rossen and Alexander W. Friedrich*

*** Correspondence:** Corresponding Author: alex.friedrich@umcg.nl

**1. Standardized paper-based questionnaire**

Collection of risk factors (to be completed personally by the employee)

(Please fill in only at the first day or when changes occur)

Name: ___________________________________________________________

Date of birth: ___________________________________________________________

Place: ___________________________________________________________

Phone number: ___________________________________________________________

Date sample taken: ___________________________________________________________

□ Earlier positive MRSA anamnesis

Profession in the hospital: □ Medical activity □ Non-medical activity

□ Intensive Care Unit (ICU)

□ Department of Burn Injuries

□ Neonatal Unit

□ Other department: __________________________________________________

□ Contact with MRSA carriers without protective clothing in the last □ 7 days □ 14 days

□ Contact with MRSA carriers with protective clothing in the last □ 7 days □ 14 days

□ Involved in home-care for relatives

□ Professional occupation (> 1 week) in a country known to be endemic for

community-associated MRSA (CA-MRSA)

□ USA

□ UK

□ Southeast Asia

□ North Africa

Skin diseases or chronic inflammatory diseases

□ Eczema (atopic eczema)

□ Paronychia (paronychia)

□ Recurrent occurring acne

□ Chronic inflammation of the outer ear or ear canal (chronic otitis externa)

□ Open wounds

□ Skin and soft tissue infection

□ Chronic inflammation of the colon (colitis, such as ulcerative colitis)

□ Chronic enteritis (Crohn's disease)

Diseases of the upper respiratory tract

□ Chronic inflammation of the sinuses (chronic sinusitis)

□ Chronic rhinitis

□ Other recurrent diseases: _____________________________________________________

□ Antibiotic treatment in the past 6 months. If so, which antibiotic: _____________________

□ Current diseases: ___________________________________________________________

Chronic underlying diseases

□ Diabetes (diabetes mellitus)

□ Other: ____________________________________________________________

□ Hospital stay (> 24 hours) within the last □ 6 months □ 12 months

□ In a German hospital

□ In a foreign hospital. If so, which country: _______________________________

□ On an Intensive Care Unit (ICU)

□ Contact with MRSA carriers in the domestic environment

□ Contact with farm animals. If so, which animals: __________________________________

□ Contact with pets. If so, which pets: ____________________________________________

□ Contact with companion animals (e.g., horses). If so, which animals: __________________

□ Contact with animal products (raw meat, such as minced pork, ham, sausage spread, etc) in

the past 12 hours
